# Instructions for authors (www.scielo.br/spmj)

**DOI:** 10.1590/S1516-31802010000200016

**Published:** 2010-03-04

## Indexing and scope

The São Paulo Medical Journal/Evidence for Health Care was founded in 1932. Its articles are indexed in Medline, Lilacs, SciELO, Science Citation Index Expanded, Journal Citation Reports/Science Edition (ISI) and EBSCO Publishing.

Published bimonthly by the Associação Paulista de Medicina, the journal accepts articles in the fields of clinical health science (internal medicine, gynecology and obstetrics, mental health, surgery, pediatrics and public health). Articles will be accepted in the form of original articles (clinical trials, cohort, case-control, prevalence, incidence, accuracy and cost-effectiveness studies and systematic reviews with or without meta-analysis), narrative reviews of the literature, case reports, short communications and letters to the editor. Papers with a commercial objective or experimental studies on animals will not be accepted.

## The Journal's policy and procedures

After receipt of the article by the Scientific Publications Sector, the authors will be provided with a protocol number. This number serves to maintain good understanding between the authors and the Scientific Publications Sector. Following this, the article will be read by the Editor, who will verify whether it is consonant with the journal's policy and interests, i.e. whether the research or review is within the fields of health or public health.

Next, the Scientific Publications Sector will verify whether the text complies with the journal's Instructions for Authors. If the text is incomplete or if it is not organized as required, the authors will be asked to resubmit their text after resolving such problems. When its format is acceptable, the Scientific Publications Sector will submit the manuscript to closed review, in which the reviewers will not sign their verdict and will not know the names of the authors. Each paper will be reviewed by at least three reviewers: one expert in the field, one associate editor (who will evaluate the article from the perspective of the reader) and one ad hoc editorial advisor (who will assess methodological aspects of the study).

The authors will then receive the reviewers’ evaluation and will be asked to resolve all the problems that have been pointed out. Once the Scientific Publications Sector receives the manuscript again, the text will be sent to the scientific editor and the proofreader, who will point out problems with sentence construction, spelling, grammar, bibliographical references and other matters. The authors should then provide all further information required.

When the text is considered acceptable for publication, and only then, it will enter the queue for publication. The Scientific Publications Sector will provide a proof, including any tables and figures, for the authors to approve. No article is published without this last procedure.

## Instructions for authors

### General guidelines: for all types of articles

Texts must be submitted exclusively through the internet. The submission system is available at http://www.spmj.hemeroteca.com.br.

The manuscript must be submitted in English. Nonetheless, it must also include a summary and five key words both in Portuguese (or Spanish) and in English. The key words must be selected from the DeCS and MeSH lists only, as explained in detail below (no other key words will be accepted).

Papers submitted must be original and the authors need to declare that the text has not been and will not be submitted for publication in any other journal. Papers involving human beings (individually or collectively, directly or indirectly, totally or partially, including the management of information and materials) must be accompanied by a copy of the authorization from the Research Ethics Committee of the institution in which the experiment was performed.

All papers submitted must comply with the editorial standards established in the Vancouver Convention (Uniform Requirements for Manuscripts Submitted to Biomedical Journals)^1^ and the quality statements for reports on clinical trials,^2^ systematic reviews (meta-analyses),^3^ and observational studies.^4^ The style known as the “Vancouver Style” is to be used not only for the format of the references, but also for the whole text. The Editors recommend that authors should familiarize themselves with this style by accessing www.icmje.org.

Abbreviations must not be used, even those in common use. Drugs or medications must be referred to using their generic names, avoiding unnecessary mention of commercial or brand terms. Any product cited in the Methods section, such as diagnostic equipment, tests, reagents, instruments, utensils, pros-theses, orthoses and intraoperative devices must be described together with the manufacturer's name and place (city and country) of manufacture in parentheses. Medications administered must be described using their generic names (not brand names) followed by the dosage used and posological data.

Grants, bursaries and any other financial support for studies must be mentioned separately, on the first page. Acknowledgements, if necessary, must be placed after the references.

For any type of study, all statements in the text that are not results from the study presented for publication in the São Paulo Medical Journal/Evidence for Health Care, but are data from other studies already published elsewhere must be accompanied by citations of the pertinent literature.

## Format

### First page (cover page)

The first page must contain: 1) the type of paper (original article, review or updating article, short communication or letter to the editor); 2) the title of the paper in English and Portuguese (or Spanish), which must be short but informative; 3) the full name of each author (do not abbreviate), his/her highest academic title attained and the institution where he/she works; 4) the place where the work was developed.

### Second page: abstract (English and Portuguese [or Spanish]) and key words

The second page must include the title, an abstract (English and Portuguese [or Spanish])^5^ structured in parts in accordance with the classification of the article (maximum of 250 words). For experimental articles, there are five items: 1) context and objective; 2) design and setting (where the study was performed); 3) methods (described in detail); 4) results; and 5) conclusions.

The abstract (English and Portuguese [or Spanish]) should contain five key words. The English terms must be chosen from the Medical Subject Headings (MeSH) list of Index Medicus, which is available on the internet (http://www.ncbi.nlm.nih.gov/sites/entrez?db=mesh).^6^ The Portuguese [or Spanish] terms must be chosen from the Descritores em Ciências da Saúde (DeCS), developed by Bireme, which is available on the internet (http://decs.bvs.br/).^7^

### References

The references (in the “Vancouver style”, as indicated by the International Committee of Medical Journal Editors, ICMJE) should be laid out in the final part of the article and numbered in the order of citation. References cited in the legends of tables and figures must maintain sequence with the references cited in the text. All the authors must be listed if there are less than six; if there are six or more, the first three should be mentioned and followed by “et al.” For books, the city of publication and the name of the publishing house are mandatory. For texts published on the internet, the complete uniform resource locator (URL) or address is necessary (not only the main home page link), so that by copying the complete address into their computer browsers, the journal's readers will be taken to the exact document cited, and not to a general website. The following are some examples of the most common types of references:

### Article in journal

– Lahita R, Kluger J, Drayer DE, Koffler D, Reidenber MM. Antibodies to nuclear antigens in patients treated with procainamide or acetylprocainamide. N Engl J Med. 1979;301(25):1382-5.

### Chapter of book

– Reppert SM. Circadian rhythms: basic aspects and pediatric implications. In: Styne DM, Brook CGD, editors. Current concepts in pediatric endocrinology. New York: Elsevier; 1987. p. 91-125.

### Text on the internet

– Morse SS. Factors in the emergence of infectious diseases. Available from: http://www.cdc.gov/ncidod/EID/eid.htm. Accessed in 1996 (Jun 5).

### Last page

The last page must contain: 1) the date and place of the event at which the paper was presented, if applicable, such as congresses or dissertation or thesis presentations; 2) sources of support in the forms of finance, equipment or drugs, and the grant numbers; 3) description of any conflicts of interest held by the authors; 4) the complete address, e-mail and telephone number of the author to be contacted for publication in the Journal.

### Figures and tables

Images must have good resolution (minimum of 300 DPI) and be recorded in “.jpg” or “.tif” format. Do not attach images inside Microsoft PowerPoint documents. If photographs are inserted in a Microsoft Word file, the images should also be sent separately. Graphs must be prepared in Microsoft Excel (do not send them in image formats) and must be accompanied by the tables of data from which they have been generated. The number of illustrations must not exceed the total number of pages minus one.

All figures and tables must contain legends or titles that precisely describe their content and the context or sample from which the information was obtained (i.e. what the results presented are and what the kind of sample or setting was). The legend or title sentence should be short but comprehensible without depending on reading the article.

São Paulo Medical Journal/Evidence for Health Care is for now published in black-and-white. Photographs, photomicrographs, bar and line graphs and any image to be published must be prepared considering that there will be no color differentiation (any color information will be discarded). Shades of gray and printing patterns (dots, stripes and others) should be used instead, with good contrast.

### Original articles

Clinical trials, cohort, case-control, prevalence, incidence, accuracy and cost-effectiveness studies, and systematic reviews with or without meta-analysis are considered original articles.

The São Paulo Medical Journal/Evidence for Health Care supports the clinical trial registration policies of the World Health Organization (WHO) and the International Committee of Medical Journal Editors (ICMJE) and recognizes the importance of these initiatives for registration and international dissemination of information on randomized clinical trials, with open access. Thus, from 2008 onwards, clinical research papers are accepted for publication only if they have received an identification number from one of the clinical trial registers that have been validated in accordance with the criteria established by WHO and ICMJE. Authors of randomized clinical trials must thus register their studies before submitting them for publication in São Paulo Medical Journal/Evidence for Health Care. The addresses for these registers are available from the ICMJE website (http://www.icmje.org/). The identification number should be declared at the end of the abstract.

Original articles must be structured so as to contain the following parts: Introduction, Objective, Methods, Results, Discussion and Conclusion. The text must not exceed 5,000 words (excluding tables, figures and references), from the introduction to the end of the conclusion, and must include a structured abstract with a maximum of 250 words.^5^ “Structured abstract” means that the abstract must contain the following items: Context and objective, Design and setting, Method, Results and Conclusion.

The structure of the document should follow the format laid out below:

1. *Title and abstract*: the study design and/or the way participants were allocated to interventions, for example “randomized” or “retrospective” study, should be mentioned in the title and in the abstract. The abstract should provide a summary of what was done and what was found.
2. *Introduction:* specify the reasons for carrying out the study, describing the present state of knowledge of the topic. Describe the scientific background or “the state of the art”. Do not include here any results or conclusions from the study. Use the last paragraph to specify the principal question of the study, and the principal hypothesis tested, if there is one. Do not include discussions about the literature in the introduction; the introduction section should be short.
3. *Objective:* describe briefly what the main objective of the study was. Clearly describe the pre-specified hypotheses.
4. *Methods*
  4.1) *Type of study:* describe the design of the study and specify, if appropriate, the use of randomization, blinding, diagnostic test standards and the time direction (retrospective or prospective). For example: “randomized clinical trial”, “double-blind, controlled placebo trial” or “accuracy study”.
  4.2) *Sample: participants or patients*: describe eligibility criteria for participants (inclusion and exclusion criteria), sources and the selection procedures. In case-control studies, describe the rationale for choosing the cases and controls, and the matching criteria. Describe the number of patients at the beginning and end of the study.
  4.2) *Setting:* indicate where the study was carried out, including the health-care ranking (for example: primary or tertiary; private or public institution). Avoid stating the name of the institution where the study was carried out (for blinding purposes). Describe only the type of institution, for example: university or public hospital.
  4.4) *Procedures (intervention, diagnostic test or exposure*, if necessary): describe the principal characteristics of any intervention, including the method, the timing and the duration of its administration or of data collection. Describe the differences in interventions administered to each group (if the study is controlled).
  4.5) *Main measurements, variables and outcome*: describe the method of measuring the primary result, in the way in which it was planned before data collection. State what the primary and secondary outcomes are. For each variable of interest, detail the methods of assessment. If the hypothesis reported was formulated during or after data collection (and not before), this needs to be specified. Describe the methods used to enhance the quality of measurements (i.e. multiple observers, training, etc.). Explain how quantitative variables were handled in the analyses.
  4.6) *Sample size and statistical analysis*: describe the sample size calculation method, the planned statistical analysis, the statistical tests used and significance levels, and any *post hoc* analyses. Describe the methods used to control for confounding factors and explain how missing data and cases lost from the follow-up were dealt with.
  4.7) *Randomization*: describe the method used to implement the random allocation sequence (for example, numbered envelopes containing computer-generated random sequences of numbers). In addition, describe who generated the allocation sequence, who enrolled participants and who assigned participants to each group (in the case of controlled trials).
5. *Results:* describe the main results. If possible, these should be accompanied by their 95% confidence interval and the exact level of statistical significance. For comparative studies, the confidence interval must be stated for the differences between the groups.
  5.1) *Participant flow*: describe the flow of participants through each stage of the study (inclusions and exclusions), the follow-up period and the number of participants completing the study (or lost from the follow-up). Consider using a flow diagram. If there was any “intention-to-treat” analysis, describe it.
  5.2) *Deviations*: if there was any deviation in the protocol, away from what was initially planned, describe it and the reasons for it.
  5.3) *Adverse events*: describe any side effect, adverse event or complication.
6. *Discussion:* provide an interpretation of the results, taking into account the study hypotheses and objectives. Emphasize the new and important factors encountered in the study, which will form part of the conclusion. Do not repeat data presented in the introduction or results in detail. Mention any limitations of your findings that should be noted and possible implications for future research, and also describe potential bias. Report any observations from other relevant studies. State the generalizability (external validity) of the findings.
7. *Conclusions:* specify only the conclusions that can be sustained by the results, together with their clinical significance (avoiding excessive generalization). Draw conclusions based on the objectives and hypothesis of the study. The same emphasis should be placed on studies with positive and negative results.

### Short communications or case series and case reports

Short communications and case reports must be limited to 3,000 words (from the introduction to the end of the conclusion). Short communications should be structured with an Introduction, Methods, Results, Discussion and Conclusion, as for original articles. Case reports should consist of an Introduction, Case Report and Conclusion.

Both short communications and case reports must be submitted with abstracts and key words. The abstracts in short communications should be structured with Introduction, Methods, Results, Discussion and Conclusion, as for original articles. The abstracts in case reports and case series should contain Context and Case Report, with a description of the case and a pertinent discussion, and Conclusion.

São Paulo Medical Journal is only interested in publishing rare and/or instructive case reports, accompanied by a systematic search of the literature, in which the relevant studies found (based on their level of evidence) are presented and discussed^8^.

### Review articles

São Paulo Medical Journal is only interested in publishing reviews that contain a systematic search in the literature on the subject, including the main databases such as Cochrane Library, Pubmed, Embase and Lilacs. Review articles have free format, but they should comply with the Vancouver Style.^1^

Nevertheless, systematic review articles or meta-analyses must comply with the same publication guidelines as for original articles.^3^ The text must not exceed 5,000 words (excluding tables, figures and references).

### Letter to the editor

Letters to the editor may address articles published in the São Paulo Medical Journal/Evidence for Health Care publication or may deal with health issues of interest. In this category, the text has a free format, but must not exceed 500 words and five references.

## References

[1] Uniform requirements for manuscripts submitted to biomedical journals (1997). International Committee of Medical Journal Editors. Ann Intern Med.

[2] Begg C, Cho M, Eastwood S (1996). Improving the quality of reporting of randomized controlled trials. The CONSORT statement. JAMA.

[3] Moher D, Cook DJ, Eastwood S, Olkin I, Rennie D, Stroup DF (1999). Improving the quality of reports of meta-analyses of randomised controlled trials: the QUOROM statement. Quality of Reporting of Meta-analyses. Lancet.

[4] STROBE Statement – Checklist on items that should be included in reports of observational studies. http://www.strobe-statement.org/index.php?eID=tx_nawsecuredl&u=0&file=fileadmin/Strobe/uploads/checklists/STROBE_checklist_v4_combined.pdf&t=1257007091&hash=7713ea8f7f2662b288689b3dab40c1cb.

[5] Haynes RB, Mulrow CD, Huth EJ, Altman DG, Gardner MJ (1990). More informative abstracts revisited. Ann Intern Med.

[6] National Library of Medicine (1998). Medical Subject Headings: annotated alphabetic list.

[7] BVS Biblioteca Virtual em Saúde Descritores em Ciências da Saúde.

[8] Oxford Centre for Evidence-Based Medicine Levels of Evidence Oxford Centre for Evidence-based Medicine Levels of Evidence (May 2001).

